# Comprehensive Review of Research Progress on Trajectory Planning and Weld Seam Tracking in Wire Arc Additive Manufacturing

**DOI:** 10.3390/mi17060698

**Published:** 2026-06-07

**Authors:** Qiang Zhu, Zaile Huang, Huan Li

**Affiliations:** 1 School of Robotics, Guangdong Open University, Guangzhou 510091, China; qzhu@gdpi.edu.cn; 2 School of Mechanical Engineering, Yangtze University, Jingzhou 519016, China; 519016@yangtzeu.edu.cn

**Keywords:** wire arc additive manufacturing, trajectory planning, droplet transition, weld morphology, image processing, arc tracking

## Abstract

Wire arc additive manufacturing (WAAM) has emerged as a promising technology for producing large-scale metal components due to its high deposition efficiency, low material cost, and design flexibility. However, the widespread industrial adoption of WAAM is hindered by challenges in geometric accuracy, process stability, and defect control, which are closely related to two critical aspects: trajectory planning and real-time weld seam tracking. This review provides a comprehensive and critical analysis of recent advances in both fields, with an emphasis on their interconnection rather than treating them as separate research streams. Unlike existing reviews that primarily summarize path planning algorithms or image processing techniques in isolation, this paper explicitly examines the integration challenges and synergistic potential between offline trajectory optimization and online vision-based monitoring. Key topics include adaptive path strategies for sharp corners and intersections, interlayer filling methods to mitigate heat accumulation and residual stress, as well as passive and active visual sensing technologies for molten pool characterization and defect detection. The review further identifies a persistent gap in closed-loop systems that combine real-time image feedback with dynamic path replanning. Based on the analysis of representative studies, current limitations are discussed and future research directions are proposed, including the development of digital twins, multi-modal data fusion, and reinforcement learning-based adaptive control. This review offers a distinct perspective aimed at advancing intelligent, high-precision WAAM systems for complex metal components.

## 1. Introduction

Wire arc additive manufacturing (WAAM) is a technology that uses metal wire as the printing material and an electric arc as the heat source to deposit layers incrementally along a predefined trajectory [1,2,3,4]. With advantages including high deposition rates (1–10 kg/h), low material cost (wire material cost is less than 20% of that of powder), high continuity, flexibility, and mold-free operation, this technology holds significant potential for manufacturing large metal structures [5,6,7,8,9]. Arc additive manufacturing can be classified by heat source type into Gas Metal Arc Welding (GMAW), Gas Tungsten Arc Welding (TIG), and Plasma Arc Welding (PAW), with various heat sources suitable for all types of metal components.

In recent years, as metal component shapes have become increasingly complex and requirements for forming accuracy have risen significantly, arc additive manufacturing faces substantial challenges. Ensuring quality during the manufacturing process is crucial for enabling large-scale batch production of arc additively manufactured specimens [10,11]. Trajectory planning constitutes the primary and most critical step in arc additive manufacturing, influencing forming performance, geometric accuracy, and structural integrity [12,13]. Ideal trajectory planning should be based on the material properties of the additive object, treating the geometric model of the printed specimen as boundary constraints, and employing algorithms to identify the optimal printing path for interlayer filling. However, arc additive manufacturing for irregular components has become a key bottleneck hindering high-quality industrial development. This is primarily due to melt pool instability caused by abrupt path changes, as well as defect propagation from one layer to the next resulting from deposition misalignment, both of which lead to accumulating deformation errors. Additionally, issues such as substrate warpage caused by excessive material temperatures further impede the commercial maturity of trajectory planning. Proper trajectory planning not only enhances the forming quality of additive manufactured parts and improves material microstructure and properties but also reduces material costs, shortens processing time, and increases production efficiency. Therefore, successful trajectory planning for complex components is fundamental to achieving advanced additive manufacturing of sophisticated equipment.

Beyond rational trajectory planning, the quality of arc additive manufacturing also depends on real-time weld shape and droplet transition morphology during the process. Image processing and real-time tracking technologies enable continuous monitoring of weld and droplet transition characteristics, thereby effectively ensuring manufacturing quality. These technologies capture images of the molten pool, droplets, and welds during the manufacturing process, then employ image enhancement, morphological threshold segmentation, and edge contour extraction techniques to analyze dynamic changes in their morphology, dimensions, and temperature distribution. This allows precise evaluation of process stability and dimensional accuracy [14]. However, poor controllability of heat sources and irregular molten pool flow patterns caused by fluid dynamics compromise formation precision [15]. Additionally, the high-temperature operating environment and interference from dust, smoke, and intense arc radiation hinder direct monitoring of weld morphology and dimensions, complicating process analysis. Therefore, mitigating interference sources, improving the accuracy of molten pool characteristic extraction, and establishing comprehensive monitoring and evaluation systems for the entire WAAM process are critical steps toward industrial-scale and intelligent manufacturing [16].

In this paper, a comprehensive literature review is conducted on trajectory planning, real-time tracking and monitoring of the molten pool and weld seam based on image processing technology, both of which are closely related to the quality of arc additive manufacturing. The study presents representative research achievements and corresponding results in trajectory planning, image processing for molten pool and weld seam tracking, and real-time monitoring, systematically identifies existing challenges, and draws actionable conclusions. Finally, relevant future research directions are proposed. This review serves as a valuable reference and guide for advancing arc additive manufacturing technology in the production of high-end, complex components. The remainder of this review is organized as follows. Section 2 presents the current research status of trajectory planning in WAAM, with a focus on handling sharp corners, intersections, start and end points, as well as step effects and heat accumulation. Section 3 systematically reviews visual imaging and infrared thermography technologies for molten pool monitoring, weld seam tracking, and defect detection, covering both active and passive sensing methods. Section 4 provides a critical discussion on the integration of trajectory planning and real-time image processing, highlighting the existing gap in closed-loop control systems. Finally, Section 5 concludes the review with a summary of key findings and proposes future research directions, including digital twins, multi-modal data fusion, and reinforcement learning-based adaptive control. Specifically, the literature search was conducted in Web of Science, Scopus, and Google Scholar using keywords such as wire arc additive manufacturing, trajectory planning, path planning, molten pool monitoring, image processing, weld seam tracking, and defect detection, covering publications from 2017 to 2026.

## 2. Current Research Status of Arc Additive Manufacturing Trajectory Planning Technology

**Figure 1:**
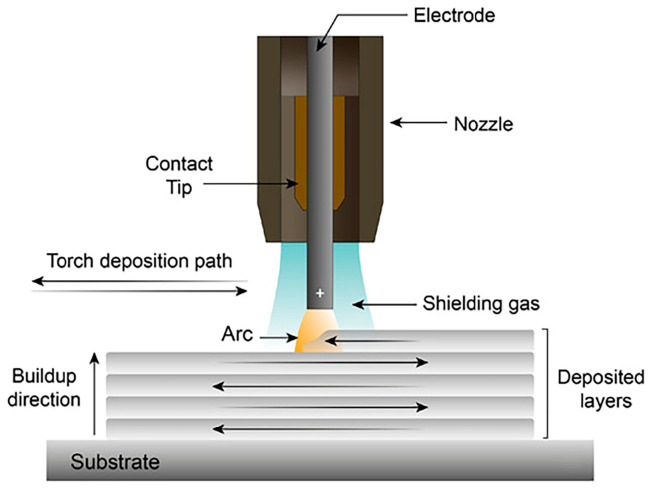
Graphic representation of WAAM operating principle [16].

Wire arc additive manufacturing is classified as an arc-based directional energy deposition process, representing a promising manufacturing technology for large-scale structures and components applicable across all industries. Figure 1 illustrates the schematic of arc additive manufacturing [16]. The quality of additive manufactured parts primarily depends on the effectiveness of trajectory planning, as this directly influences temperature distribution and residual welding stress. Relying solely on human intuition to determine deposition sequences is unreliable, particularly when processing complex and irregular components. Ideally, selecting an optimized deposition sequence can effectively mitigate overheating issues [17]. With advancements in additive manufacturing technology, trajectory planning algorithms are evolving toward greater intelligence and adaptability.

### 2.1. Path Planning

The arc additive manufacturing process primarily consists of three critical steps: three-dimensional (3D) modeling, path planning, and deposition. Path planning directly impacts printing accuracy. Arc additive manufacturing path planning mainly involves determining the slicing method for the workpiece and establishing path filling strategies within each slice [18]. Additionally, since additive manufacturing employs a bottom-up deposition approach, the slicing method between layers and the filling strategy within each layer significantly influence the dimensional accuracy of the final product. The slicing method between models is a crucial step; it enables the discretization of preform models. The most widely used model slicing methods currently involve discretization between planes, with the “plane slicing method” and “adaptive slicing method” being the most typical. For specialized workpieces, the “model surface slicing method” is employed. Dai et al. [19] noted that traditional slicing methods struggle to produce high-precision spatial surfaces and proposed that smooth surfaces be filled during rotation, whereas surfaces with inflection points should be filled using the raster method. Experimental results demonstrated superior accuracy with this approach. Furthermore, Michel et al. [20] proposed integrating feature-based design modules into conventional layer by layer strategies, dividing each layer into independent deposition segments to allow path planning tailored to the target geometry.

**Figure 2:**
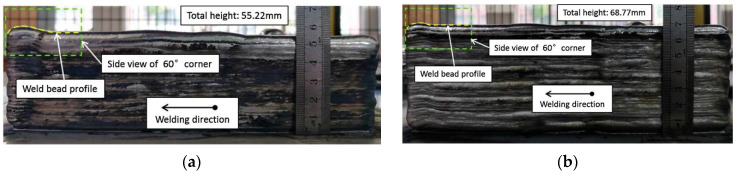
Side appearance of the finally deposited thin-walled structure. (**a**) SBM WAAM; (**b**) CCM-controlled WAAM [23].

Given the layer by layer accumulation characteristic of arc additive manufacturing, the planning of deposition paths is inherently linked to the workpiece surface. During wire arc additive manufacturing, surface morphology is influenced by corners, sharp edges, intersection points, and arc extinction points. As the printing height increases, these effects progressively intensify, ultimately leading to print failure [21,22]. Regarding corner issues in additive manufacturing, most current research approaches combine motion parameters with process parameters for coordinated control. To address self-overlapping at thin-walled structure corners (i.e., the unwanted local accumulation of filler material caused by the overlap of the deposited bead with itself when the torch decelerates or changes direction at sharp corners), Ding et al. [23] proposed a corner cross-section model that correlates the welding speed of arc additive manufacturing with the spherical molten pool at corner intersections. They demonstrated that the spherical molten pool model (CCM control strategy) effectively mitigates self-overlapping at corners, as shown in Figure 2a. Although greater structural height (or more layers) substantially reduces cumulative errors at corners (Figure 2b), the CCM-enhanced control strategy enables the production of higher-quality thin-walled structures.

**Figure 3:**
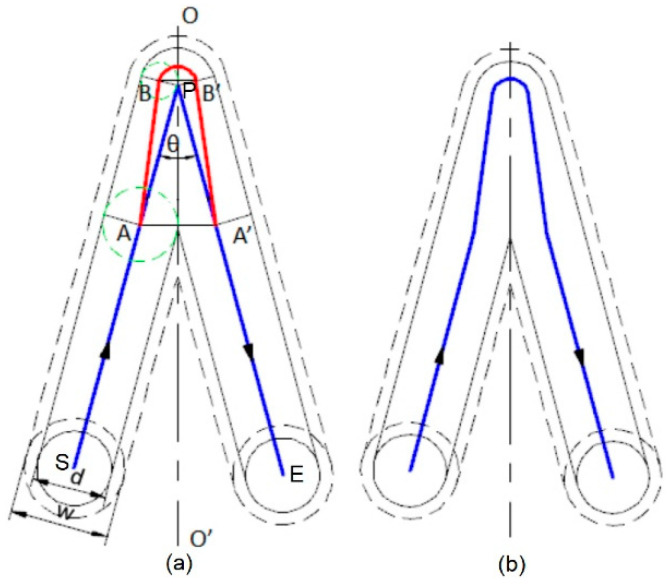
(**a**) The single bead path planning at sharp corners; (**b**) the modified corner path [24].

**Figure 4:**
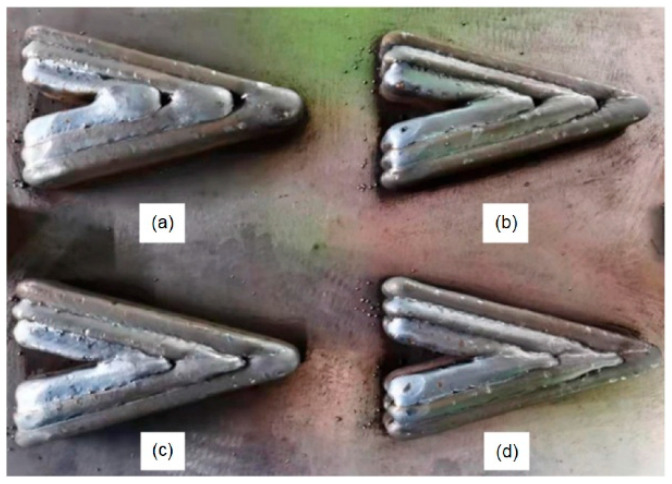
Using different methods to deposit corners. (**a**) The traditional contour paths with uniform parameters; (**b**) The proposed corner paths turning at the reference position P1with adaptive parameters; (**c**) The proposed corner paths turning at the reference position P2 with adaptive parameters; (**d**) The proposed corner paths turning at the reference position P3 with adaptive parameters [24].

To address the issues of excessive filling and self-filling at corners of thick-walled structures, Ding et al. [24] proposed an optimized corner path method for gap-free configurations, as illustrated in Figure 3. By generating multi-channel paths with varying center distances and additional gap filling paths at corners, they developed a welding parameter model using artificial neural networks (ANNs) to select optimal welding parameters for corner correction, thereby avoiding both excessive self-overlapping and insufficient filling. The results are shown in Figure 4. The deposition angle region produced using the traditional contour path (Figure 4a) exhibits overlapping traces and underfilled gaps, resulting in poor surface accuracy. Moreover, the accumulation of filling material at the corner further destabilizes the deposition layer, leading to poor formability. In contrast, the deposition angle region using the optimized path effectively mitigates both self-overlapping and underfilling issues (Figure 4b–d).

**Figure 5:**
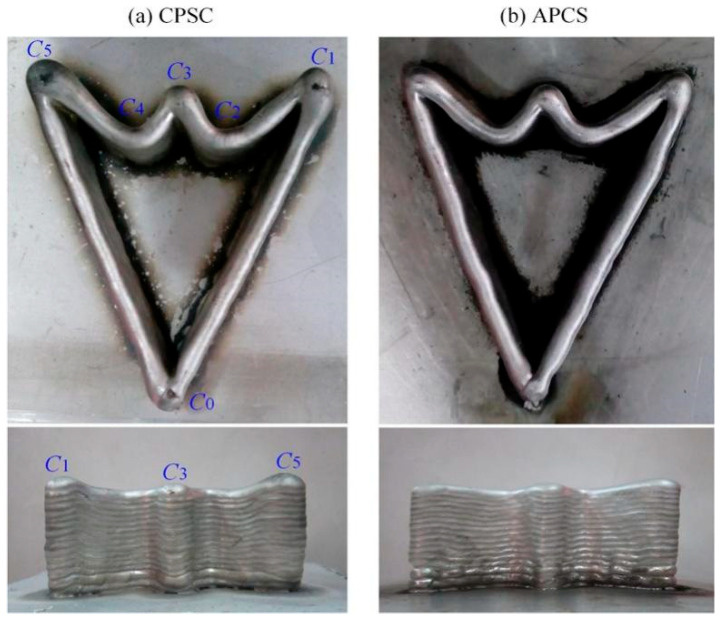
The sedimentary shape ratio between CPCS and APCS [25].

When encountering sharp corners with high curvature during WAAM, the prevailing solution adopted by most researchers involves reducing the welding robot’s speed to meet dynamic constraints; however, this approach leads to excessive filler material accumulation around the corners (i.e., hump formation). Li et al. [25] proposed an Adaptive Process Control Scheme (APCS), which divides the deposition path into multiple segments and automatically selects dynamically constrained welding and wire feed rates for each segment based on predefined process parameters. This rate matching ensures continuous and stable deposit output throughout the manufacturing process. The experimental results shown in Figure 5 demonstrate that the conventional control scheme (CPCS) exhibits significant dimensional errors in both horizontal and vertical directions at the sharp corners of positions C1, C3, and C5, along with excessive deposit overlap. In contrast, the APSC deposition model exhibits smoother horizontal progression and reduced vertical elevation variations, indicating superior deposition performance compared to the CPCS.

**Figure 6:**
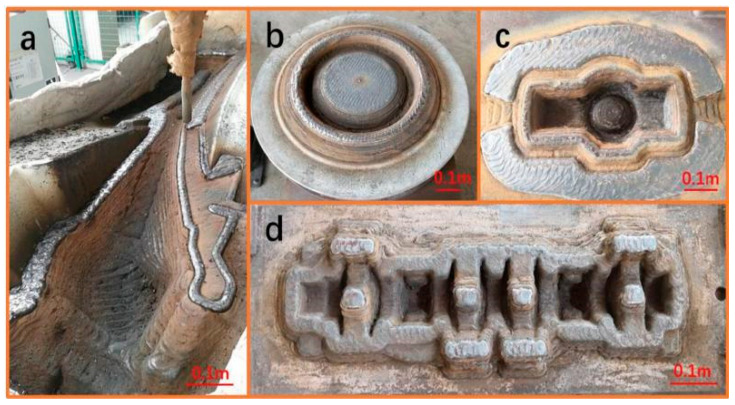
Four hot forging dies after WAAR: (**a**) blade die; (**b**) turbine disk die; (**c**) valve body die and (**d**) crankshaft die [26].

To address the issue of insufficient material filling at sharp corners, Shen et al. [26] proposed a path generation method for arc additive manufacturing used in hot forging dies. By calculating the lengths and inclination angles of each segment in the contour line, the relatively optimal scanning direction is determined, thereby reducing the likelihood of sharp corners. Based on the selected extreme point positions, the internal area is divided and the path space is adjusted to prevent bottom filling. Finally, through adaptive adjustments, overlapping weld lines are eliminated, resulting in reduced material accumulation. The actual printed sample of the arc additive manufacturing process is shown in Figure 6, demonstrating that the printed parts with this optimized path exhibit superior surface quality.

**Figure 7:**
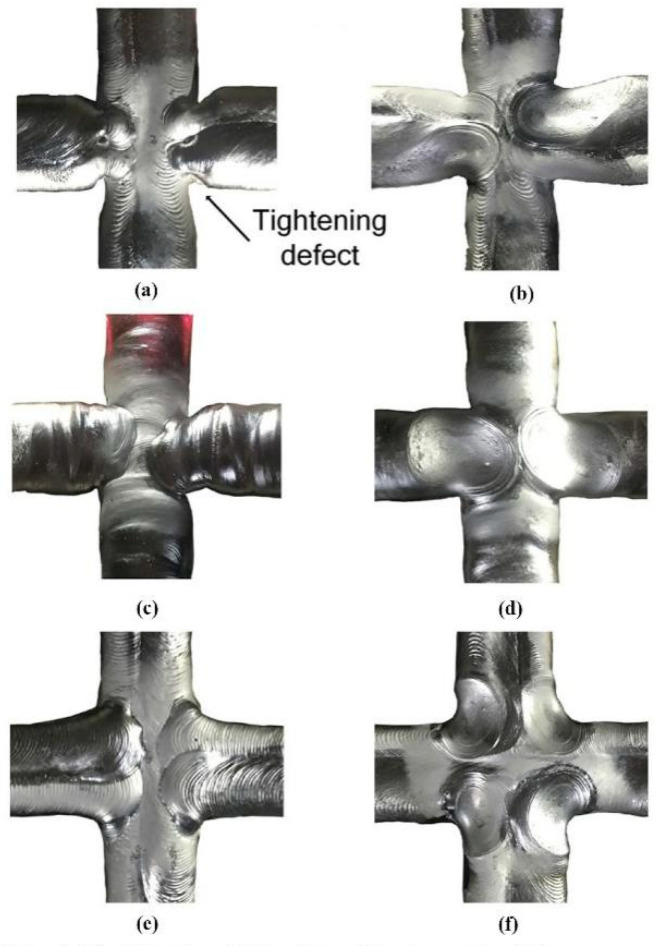
Surface contour images of three sedimentation strategies. (**a**) Parallel and arc striking; (**b**) parallel and arc extinguishing;(**c**) oscillation and arc striking; (**d**) oscillation and arc extinguishing; (**e**) ELE and arc striking; (**f**) ELE and arc extinguishing [27].

Path intersections during additive manufacturing can lead to localized filler material accumulation, resulting in weld beads. Current solutions primarily focus on minimizing path repetition, but this approach often introduces defects such as weld beads or arc pits at intersection points. To address this, Li et al. [27] proposed a novel path strategy called the End-Lateral Extension Path Strategy (ELE), offering an alternative for non-intersecting paths in arc additive manufacturing. The printed object using this path is shown in Figure 7. Compared to conventional path planning methods, the ELE approach eliminates the persistent issue of defects at intersections, achieves more consistent interlayer height increments, and improves profile quality with reduced fitting plane deviation.

**Figure 8:**
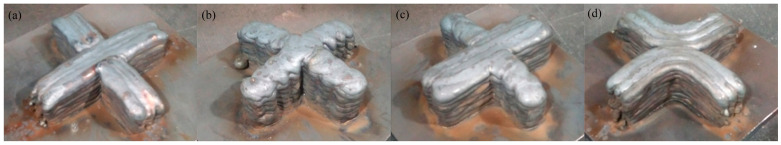
X-shaped crossing paths were fabricated using different strategies. (**a**) Cross overlap; (**b**) cross-wave overlap; (**c**) Wave overlap; (**d**) Single overlap [28].

Veiga et al. [28] investigated X-shaped cross paths and fabricated SS316L-Si stainless steel arc additive manufacturing specimens using four distinct path configurations: cross-overlapping, cross-wavy, wavy-overlapping, and single-overlapping, as shown in Figure 8. The parts fabricated via cross-overlapping (Figure 8a) exhibit geometric similarity to those produced by the single-overlapping method (Figure 8d). While cross-wavy-overlapping (Figure 8b) optimizes material utilization, the wavy trajectory induces wall fluctuations that may complicate machining processes. The continuous wavy-overlapping approach (Figure 8c) requires additional waveforms to ensure proper integration into the near-net-shape (NNS) structure. Therefore, for components with X-shaped crossings, the cross-overlapping and cross-wavy strategies represent the optimal approaches (Figure 8d).

**Figure 9:**
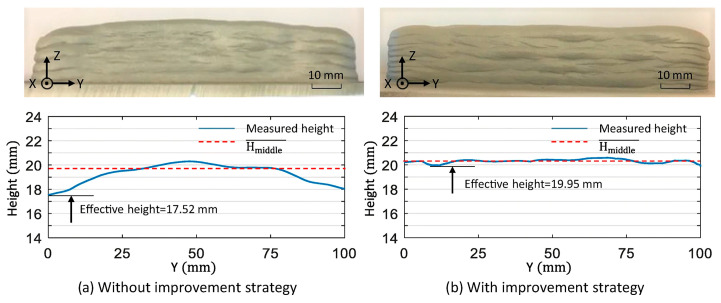
Thin-wall parts and the corresponding height measurements. (**a**) Without improvement strategy; (**b**) With improvement strategy [29].

To address the issue of irregular weld seam geometry caused by the arc initiation point, Wang et al. [29] proposed an improved strategy comprising varying travel speeds and additional return paths. When an alternating-direction path (where adjacent layers are deposited in opposite directions) is employed to compensate for the start hump and end crater, the accumulation at both ends of the weld seam remains lower than in the central section (Figure 9a), resulting in limited improvement in height error at the seam ends. However, with the improved strategy, significant reduction in end height deviations is observed, effectively enhancing the geometric accuracy of the part, as shown in Figure 9b.

To investigate the impact of weld beads generated at the arc extinction point on interlayer transitions during additive manufacturing, Wang et al. [30] proposed an arc additive manufacturing path planning method based on the water-reflow rule, ensuring structural density while enhancing path planning responsiveness. This approach distributes the computational cost of generating all paths in a single operation, improving real-time performance and dynamism of path planning. Throughout the process, path directions between adjacent layers are adjusted only when an arc extinguishes, thereby enhancing formation stability and deposition efficiency. Giordano et al. [31] introduced a “continuous tool path scalar thermal field” strategy designed to create continuous deposition paths, reduce arc extinction cycles, and improve part quality.

**Figure 10:**
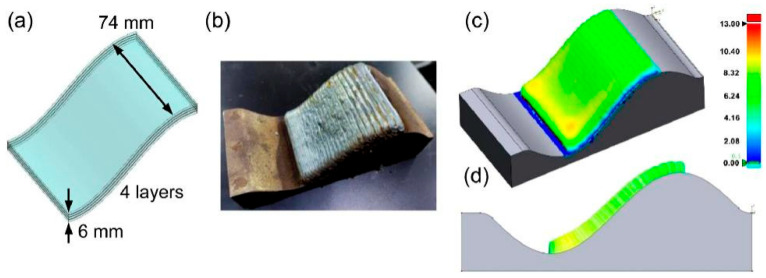
Multi-layer CL-WAAM: (**a**) Four-layer curved surface model; (**b**) deposited part with curved layers; (**c**) appearance and thickness distribution; (**d**) thickness distribution of longitudinal-section [33].

**Figure 11:**
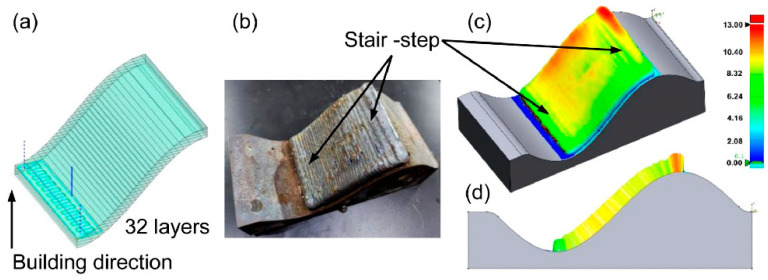
Multi-layer FL-WAAM: (**a**) Flat layer slicing model; (**b**) deposited part with flat layers; (**c**) appearance and thickness distribution; (**d**) thickness distribution of longitudinal-section [33].

During the path planning process for arc additive manufacturing, factors such as step effects between layers, travel path planning for the welding torch within each layer, filling methods, as well as heat accumulation, residual stresses, and warping deformation during printing must be considered. To address the step effect generated during additive manufacturing, current solutions employ an adaptive slicing approach, where the elevation of each layer is adjusted based on the geometric characteristics of the preceding layer to minimize its impact on final product quality. When the height difference between adjacent slicing layers exceeds a predefined threshold, the height is reduced; otherwise, it is increased [32]. Hu et al. [33] proposed a region-based path planning method (CL-WAAM) and compared it with the traditional FL-WAAM method. As shown in Figure 10, four curved printed layers were deposited using CL-WAAM on a curved substrate, yielding an average thickness of 7.81 mm with a standard deviation of 1.24 mm and a tolerance of 1.81 mm. In contrast, Figure 11 demonstrates that the FL-WAAM method deposited 32 layers of planar material from the substrate base along the building direction, employing both zigzag paths within internal regions and contour-enveloping paths along the outline. The printed welds exhibited an average thickness of 8.94 mm, 62.4% greater than that of CL-WAAM, and showed uneven thickness distribution at the top surface, particularly at the bottom and top due to pronounced step effects caused by material stacking in planar layers, indicating that the CL-WAAM strategy effectively mitigates step effects. Furthermore, Zhang et al. [34] developed a composite filling algorithm that fills internal regions through linear scanning and fills external contours via offset filling. Experimental results demonstrate that this method effectively addresses the collapse issue caused by the step effect.

**Figure 12:**
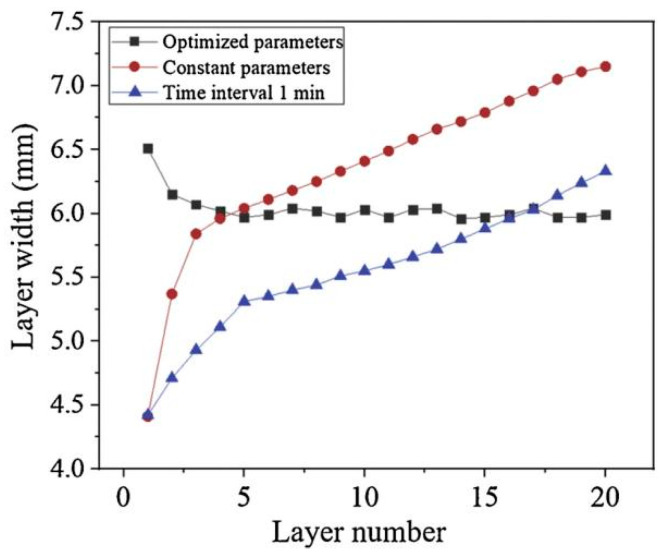
Comparison of the layer width using different deposition strategy [35].

To investigate the impact of travel path planning on the arc additive manufacturing process, Zhao et al. [35] abandoned conventional approaches that incorporated idle time and active workpiece cooling. Instead, they proposed a process planning strategy based on thermal behavior considerations, dividing the deposition process into distinct stages and employing a finite element model to analyze typical heat transfer cycles for predicting interlayer temperatures and thermal deformation in subsequent layers. Their findings revealed that the large shell-shaped component comprising 753 layers achieved superior dimensional accuracy compared to other path planning methods. The variance in optimized layer width was 0.1178 mm, whereas that of other layer widths was 0.6399 mm and 0.4868 mm, respectively (Figure 12). Additionally, the arc additive manufacturing time under the optimized method was reduced by at least 20 min compared to conventional methods, demonstrating its effectiveness in enhancing manufacturing efficiency.

### 2.2. Current Research Status of Path Filling Technology

**Figure 13:**
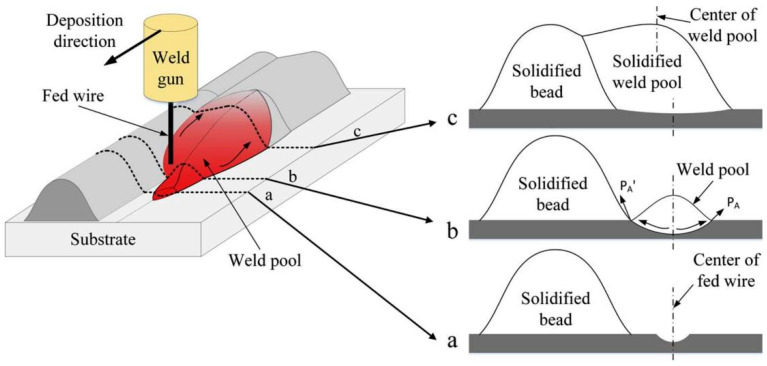
The overlapping process of melting weld beads. (**a**) The position of thefedwire; (**b**) The position that the weld pool touches the already deposited neighboring bead; (**c**) The position that the weld pool solidifies [36].

The filling strategy planning for arc additive manufacturing involves layer by layer filling after processing model slices. The most widely used filling strategies fall into four categories: contour offset method, reciprocating linear method, combined contour offset and reciprocating linear method, and partition method (array filling method). Regardless of the chosen filling approach, establishing an overlapping model (BOM) is essential; however, existing models only account for the geometric areas of adjacent welds while neglecting the extension of the molten weld. To address this, Li et al. [36] developed an enhanced bead overlapping model (E-BOM). As shown in Figure 13, the study employed an artificial neural network to predict weld offset distances and a reasoning algorithm to calculate the center-to-center distances between adjacent deposition paths. The results demonstrated that this model improves the surface flatness of the formed part and reduces internal defects.

**Figure 14:**
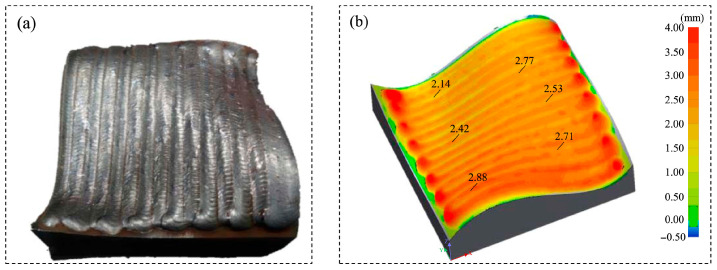
The experimental result of single-layer multi-bead deposition on S2: (**a**) Shaped morphology; (**b**) contour map of thickness distribution [37].

Current research on interlayer filling planning algorithms primarily focuses on path filling strategies for planar layers. Path planning algorithms for planar layers are mainly categorized into five types based on their origin and development: raster method, contour method, spiral method, fractal method, and composite method. Among these, research on path planning algorithms for curved surface layers with arbitrary curvature variations remains limited. To address this, Ni et al. [37] developed an additive manufacturing method for complex surfaces to overcome forming challenges caused by varying welding position effects. The method employs response surface methodology to statistically model the relationship between process parameters and droplet response characteristics, systematically investigating forming parameters and their impacts on part geometry dimensions and formability, ultimately achieving stable and consistent droplet deposition across all surface angles and orientations. Experimental results shown in Figure 14 demonstrate consistent weld dimensions without significant segmentation between adjacent welds, smooth surface transitions, and grain surface dimensional deviation and non-uniformity index below 1.55%. The overall deposition thickness uniformly ranges from 2.4 mm to 2.9 mm, with maximum variations in peak and valley heights between adjacent welds measuring only 0.5 mm.

Regarding filling methods in additive manufacturing processes, Vo et al. [38] categorize them into two main types: path planning approaches for continuous path filling and array filling algorithms. The former primarily includes parallel-line filling paths, contour-bias filling paths, and hybrid filling paths; the latter mainly comprises scan-line filling algorithms, boundary-marking filling algorithms, seed filling algorithms, and scan-line seed filling algorithms. However, a single filling method alone cannot achieve optimal results. Consequently, the integration of multi-degree-of-freedom approaches and diverse methods for filling non-uniform parts represents the future trend.

**Figure 15:**
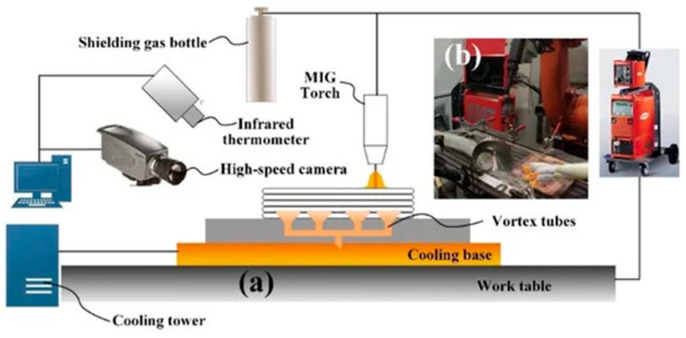
WAAM deposition system: (**a**) schematic diagram; (**b**) physical diagram [39].

Most researchers have pointed out that heat accumulation caused by filler material deposition during WAAM can be reduced by adjusting process parameters. However, such solutions are often complex and require extensive experimentation to determine optimal parameters. In response, Wang et al. [39] proposed a novel online eddy current cooling system, as shown in Figure 15. By installing eddy current tubes and a water-cooled base on the arc additive manufacturing system, an air compressor generates high-pressure airflow within the tubes to facilitate heat transfer. As low-pressure, high-temperature gas is expelled from both ends of the tubes, low-pressure, low-temperature gas is immediately produced. Experimental results demonstrate that this device effectively mitigates common defects such as reduced forming accuracy, coarser grain structure, and porosity caused by heat accumulation. Koazmernik et al. [40] developed an arc additive manufacturing system based on a three-axis robot. Infrared thermometers are used to monitor the final deposition layer, and a control system halts deposition after each layer to maintain interlayer temperatures below a predetermined threshold, ensuring proper cooling and consistent deposition shape stability while minimizing heat accumulation.

**Figure 16:**
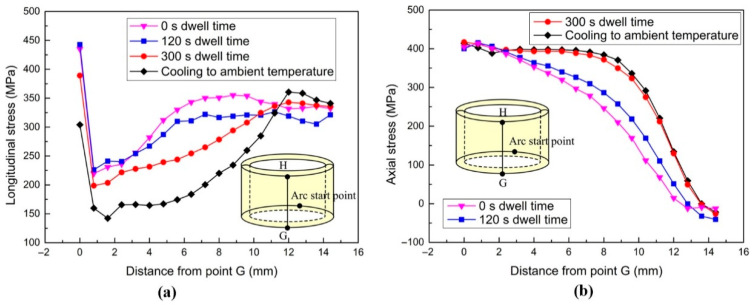
Residual stress distribution along the GH line. (**a**) Longitudinal stress; (**b**) axial stress [41].

**Figure 17:**
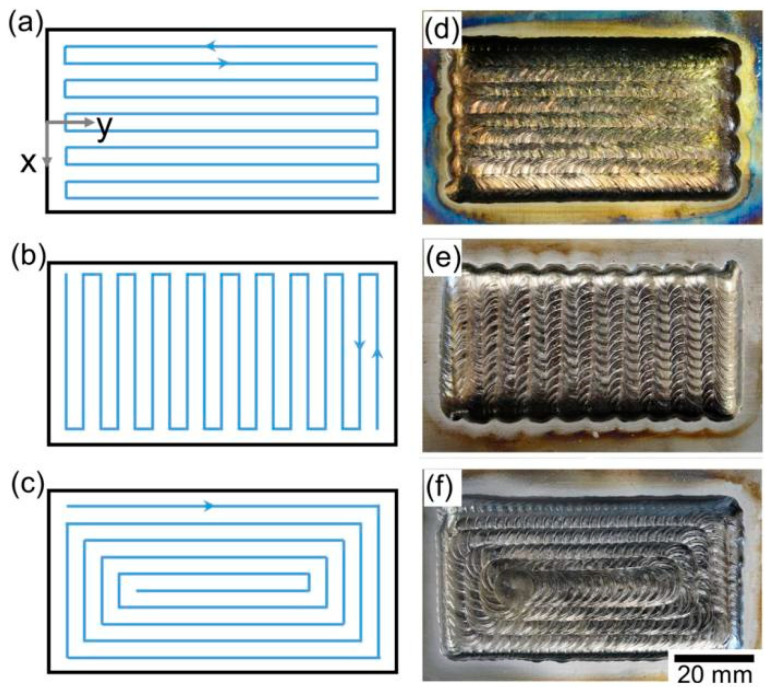
Different deposition strategies for rectangular structures: (**a**) Long deposition strategy; (**b**) short deposition strategy; (**c**) spiral deposition strategy; (**d**–**f**) corresponding morphologies of the as-deposited samples, respectively [43].

**Figure 18:**
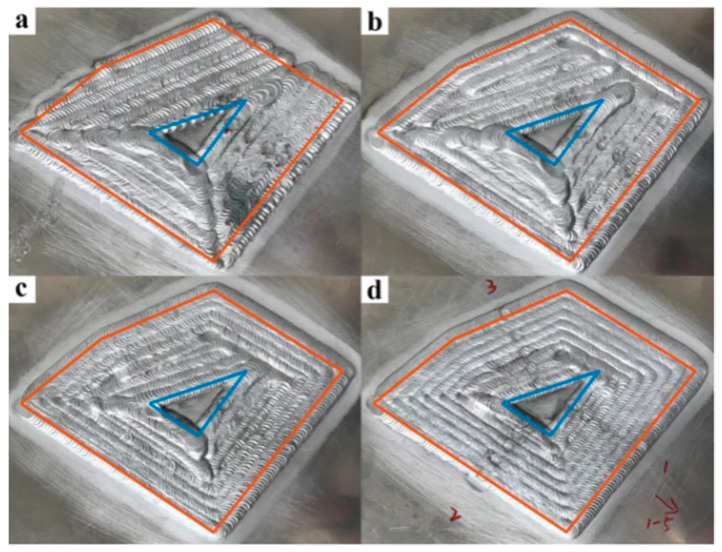
WAAM sedimentation effect based on CMT. (**a**) Sample 1; (**b**) Sample 2; (**c**) Sample 3; (**d**) Sample 4 (the inner and outer contours of the designed target shape are represented by blue and orange lines, respectively [44]).

To investigate the formation mechanism of residual stresses during additive manufacturing, Li et al. [41] demonstrated that interlayer residence time significantly influences workpiece residual stresses. By establishing a thermomechanical coupled finite element model, they evaluated the distribution of transverse and longitudinal residual stresses under varying interlayer residence times, revealing that increased residence time leads to more uniform stress cycle curves and diminished interlayer stress release effects (Figure 16). Additionally, Huang et al. [42] highlighted the substantial impact of thermal conditions on residual stresses under different scenarios. Experimental and numerical analyses revealed that identical deposition orientations generate greater temperature gradients and result in more pronounced stress deformation during solidification. Wu et al. [43] employed three identical rectangular deposits using long, short, and spiral deposition strategies, followed by 800 s of cooling post-deposition. Residual stress measurements via drilling on three identical components showed maximum stress accumulation in spiral-pattern deposits, followed by long deposition strategies, with minimal stress in short deposition configurations (Figure 17). Zhang et al. [44] developed a filling planning strategy combining zigzag and contour offset techniques (Figure 18). Comparative analysis of four printed samples demonstrated that offsetting the external contour prior to the internal dimensions of zigzag fills optimizes residual stress distribution and contour accuracy in arc additive manufacturing layer construction.

**Figure 19:**
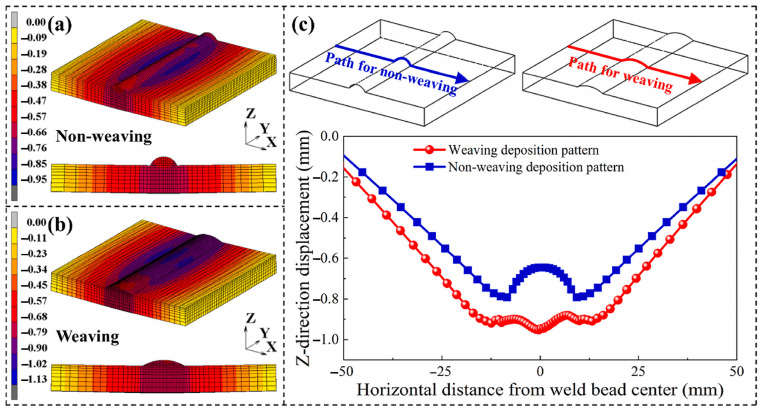
Warping deformation distributions for the non-weaving and (**a**) weaving; (**b**) deposition patterns, and (**c**) their Z-direction displacement variations in nodes on the center surface paths [45].

To investigate warpage deformation caused by heat accumulation during additive manufacturing, Zhao et al. [45] developed four distinct deposition sequence strategies (in–out and out–in) and deposition direction configurations (alternating and consistent), analyzing their effects on warpage deformation using a thermomechanical coupled finite element method. Compared to non-woven patterns, woven deposition patterns exhibit more uniform deformation and lower residual stresses due to wider and shallower melt pools, as shown in Figure 19. Comparative results revealed that the in–out deposition sequence achieves faster heat dissipation and a more uniform temperature field during the cooling phase, thereby reducing both warpage deformation and residual stresses. Furthermore, all alternating deposition direction strategies demonstrated uniform deformation, providing a solid basis for the woven manufacturing process in arc additive production of disk-shaped components.

A comprehensive comparison table (new Table 1) have been added. This table systematically compares the representative trajectory planning methods discussed in the review across five dimensions: method category, specific technique, applicable component geometry, key advantages, and main limitations. The table provides readers with a clear and concise overview of the strengths and weaknesses of different approaches, facilitating informed selection for specific WAAM applications.

## 3. Current Research Status of Real-Time Weld Seam Tracking

The dynamic tracking technology for weld and droplet transition morphology serves as a critical component in ensuring additive manufacturing quality and precision. It primarily involves real-time monitoring of dynamic characteristics such as molten pool morphology, dimensions, temperature distribution, and flow behavior, combined with real-time feedback from the forming process to adjust process parameters and forming paths dynamically. This enables precise control over the molten pool state, thereby guaranteeing the geometric accuracy, surface quality, and internal structural uniformity of the fabricated parts. Image processing technology, as the core technical approach for dynamic tracking in arc additive manufacturing, leverages its non-contact and high-speed advantages. Utilizing high-resolution, high frame rate imaging systems, it captures dynamic image sequences of the molten pool and welds, facilitating continuous monitoring and online analysis of both the stability of the manufacturing process and the quality of the printed parts [46].

### 3.1. General Workflow of Image Processing for WAAM Monitoring

#### 3.1.1. Image Preprocessing

**Figure 20:**
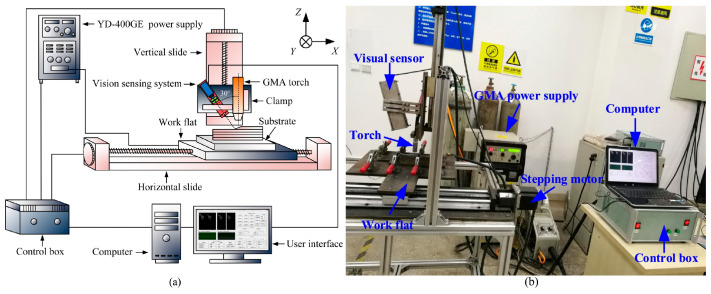
On-site Monitoring and Control System for GMA-AM. (**a**) Schematic diagram; (**b**) on-site diagram [47].

As the most widely used research technique, visual imaging technology involves analyzing pixel variations in molten pool and weld images to identify their key characteristic features. To this end, Xiong et al. [47] developed a passive visual sensing system composed of a camera and a dual-prism array, as shown in Figure 20. Through procedures including camera calibration, image correction, molten pool extraction, and edge reconstruction, the layer width is measured in real time. The study demonstrates that the passive visual sensing and feedback control system significantly enhances layer width stability, with this stability increasing as deposition depth increases. After reaching a certain number of layers, the maximum width error between welds is less than 0.5 mm.

**Figure 21:**
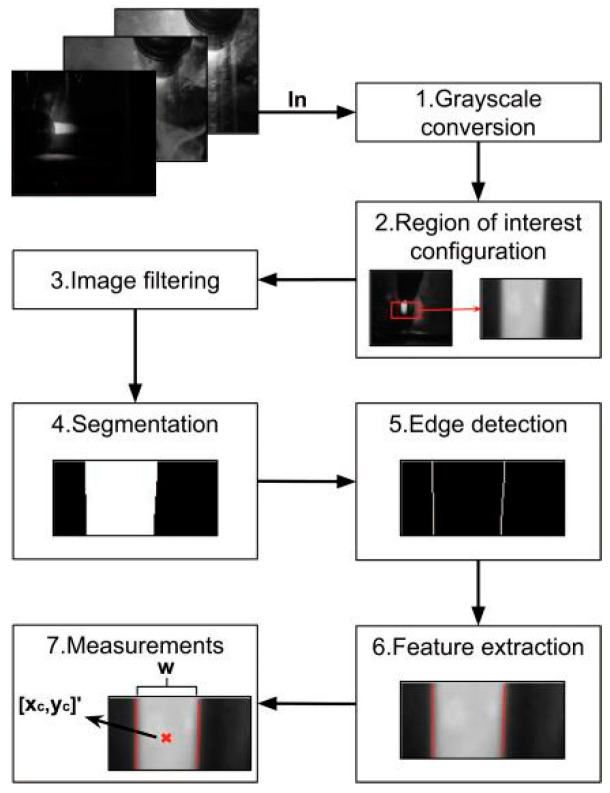
Steps of the computer vision algorithm [48].

Couto et al. [48] extracted the geometric characteristics of the melt droplets (width and centerline) using a monocular camera. The captured images were first converted to grayscale images, after which the region of interest (ROI) was defined. Adaptive thresholding was employed for image segmentation, and filtering steps were applied to reduce the low signal-to-noise ratio during the deposition process, as shown in Figure 21. Experimental results demonstrate that the proposed vision-based algorithm serves as a reliable solution for developing and implementing arc additive manufacturing monitoring systems, enabling real-time parameter adjustment during the printing process. By filtering features and measurements and utilizing the previous process state to eliminate outliers, weld width monitoring can be effectively performed even during highly noisy deposition processes such as cold metal short-circuit transition arc additive manufacturing.

#### 3.1.2. Edge Contour Extraction

Jiao et al. [49] developed a visual acquisition system using a high-speed CCD phase mechanism and employed the Sobel edge detection algorithm to extract edge contours, thereby reducing external interference from arc light on the imaging acquisition of the welding pool. Feng et al. [50] created a semi-automatic video annotation tool for arc additive manufacturing based on the Computer Vision Basic Model (SAM) and the Video Object Tracking Model (XMem). This tool annotates video frames hundreds of times faster than traditional manual methods, enabling rapid quantitative analysis of arc additive manufacturing videos. The dynamic tracking stability of the visual annotation tool was verified through online closed-loop control of wire position, analysis of droplet transfer behavior, and the assembly of a dataset using a specialized deep learning segmentation model.

**Figure 22:**
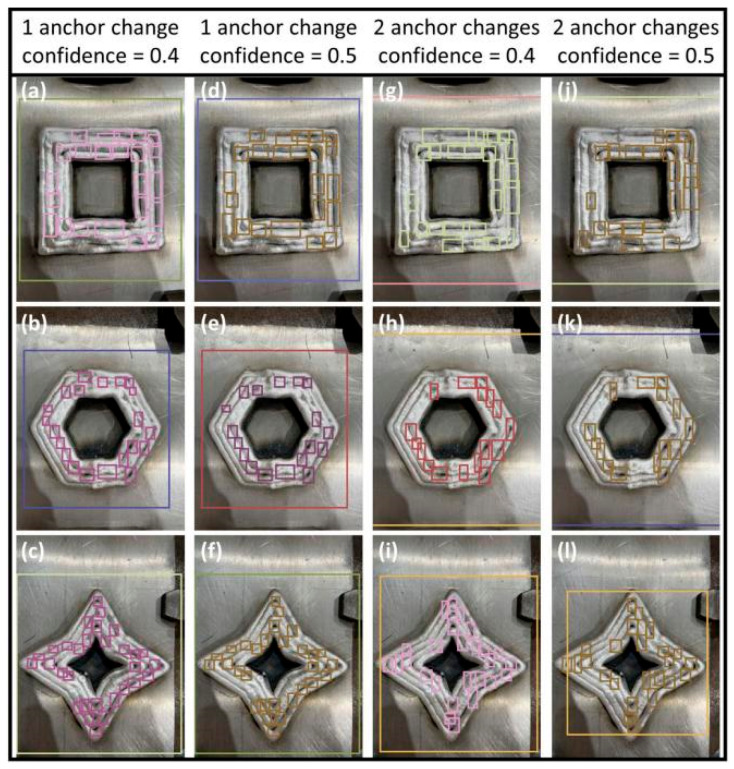
Performances comparisons on 1-anchor-change and 2-anchor-change. (**a**–**c**): original anchor setting (Group A); (**d**–**f**): 1-anchor-change (Group B); (**g**–**i**): 2-anchor-change (Group C); (**j**–**l**): 3-anchor-change (Group D) [51].

To address the issue of inaccurate defect detection (e.g., porosity) during arc additive manufacturing, Li et al. [51] developed an intelligent visual image processing system. Based on the YOLOv3 architecture, moderate adjustments to anchor point settings (as shown in Figure 22) revealed that for models with only one anchor point change, abnormal predictions were acceptable when the confidence threshold was set at 0.4 (Figure 22a–c); however, setting the threshold to 0.5 removed some low confidence bounding boxes, resulting in missed genuine anomalies (Figure 22d–f). Figure 22g–l demonstrate similar results for the two-anchor-point transformation model. It is evident that a higher confidence threshold eliminates certain bounding boxes containing internal right-side anomalies. In the two-anchor-change model, the bounding boxes consistently exceed those in the single-anchor-change model, sometimes even surpassing the image dimensions. Consequently, the model with one anchor point change and a confidence threshold of 0.4 was selected as the optimal predictor for anomaly and component identification, achieving 53% surface anomaly detection accuracy and 100% component positioning precision, a prerequisite for high-precision welding quality assessment.

#### 3.1.3. Feature Parameter Calculation

**Figure 23:**
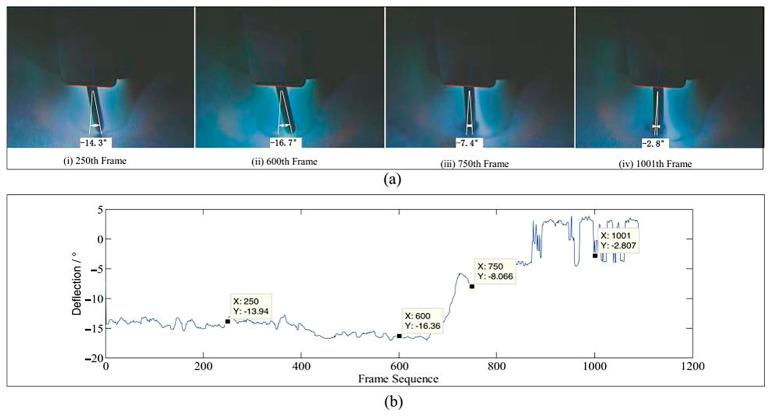
The wire deflection values retrieved manually and by software: (**a**) Manually measured result; (**b**) software-detected result [52].

To address the issues of deposition defects and dimensional errors in arc additive manufacturing caused by wire torsion during robotic arm operation, which leads to position deviation of the wire feeder, Zhan et al. [52] proposed a vision-based measurement and monitoring method and developed software for automatic wire deviation detection to enable real-time monitoring and correction of such deviations. As shown in Figure 23, to validate the software, manual measurements of wire deviation were first performed on selected images extracted from the arc additive manufacturing video recordings (Figure 23a: frames 250, 600, 750, and 1001). The software was then applied to obtain wire deviation results from the same video (Figure 23b). Comparison of manual measurements with software results revealed that the maximum deviation between the two sets was less than 1 degree, confirming the effectiveness of both the software and the wire deviation correction method.

### 3.2. Sensor Specific Image Processing Characteristics

#### 3.2.1. Processing of Infrared Thermal Images

The application of infrared thermal imaging technology for dynamic monitoring in arc additive manufacturing involves utilizing the high-temperature thermal radiation characteristics of the molten pool. Infrared cameras capture the molten pool’s thermal signals and convert them into thermal images, enabling real-time tracking of the molten pool’s contour and temperature distribution. This ensures the stability of the deposited layer and thereby improves the dimensional accuracy of arc additive manufactured components. To address this, Yu et al. [53] proposed an infrared temperature measurement and deep learning-based coating offset monitoring technique. The method first establishes a temperature field measurement system for arc additive manufacturing using infrared thermal imaging cameras, identifies a region of interest (ROI) on the side wall of the additive sample, and employs an optimized directional histogram (HOG) algorithm to extract temperature distribution patterns of the deposited layer. The study also analyzes the separability of welding side wall temperature fields under varying offset conditions. Finally, the temperature images from the ROI are used as input to construct a coating offset recognition model via convolutional neural networks (CNNs). Experimental results demonstrate that the model achieves an accuracy rate exceeding 99% in coating offset identification, effectively overcoming the arc light interference challenge inherent in vision-based recognition methods.

**Figure 24:**
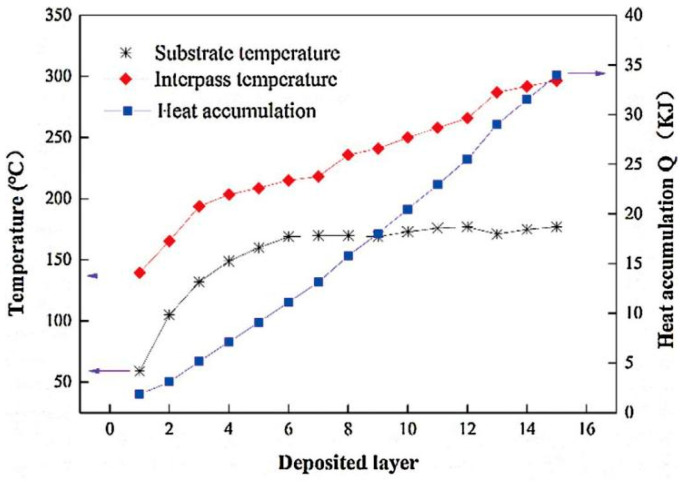
The variation in temperature and heat accumulation during fabrication [54].

To investigate the impact of thermal accumulation on the deposition stability of arc additive manufacturing samples, Wu et al. [54] directly measured the surface temperature of the deposited layer using an infrared thermal imager. As shown in Figure 24, the average temperature of the arc additive manufacturing substrate rose rapidly during the first five processes before reaching an equilibrium value. Due to the thermal accumulation effect, direct temperature measurements taken at the wall deposition points using a non-contact infrared thermal imager yielded more accurate temperature profiles than those obtained by R-type thermocouples installed at predetermined substrate positions.

**Figure 25:**
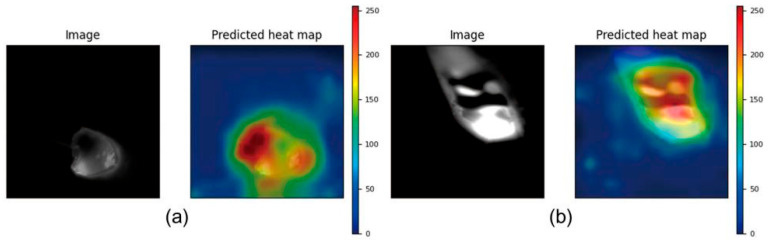
Class activation mapping results: (**a**) Visible light image; (**b**) infrared image [55].

To address the issue of insufficient detection accuracy in dynamic monitoring of arc additive manufacturing, where a single infrared camera is frequently affected by arc light and splatter interference, Wang et al. [55] developed a dual-input convolutional neural network model based on a multi-modal mutual fusion network (MMFNet), integrating feature-level visible and infrared data as shown in Figure 25. Compared to single-modal models, the dual-modal system demonstrates superior sensitivity to both visible and infrared light, achieving a prediction accuracy of 98.34%.

#### 3.2.2. Processing of Visible Light Images

**Figure 26:**
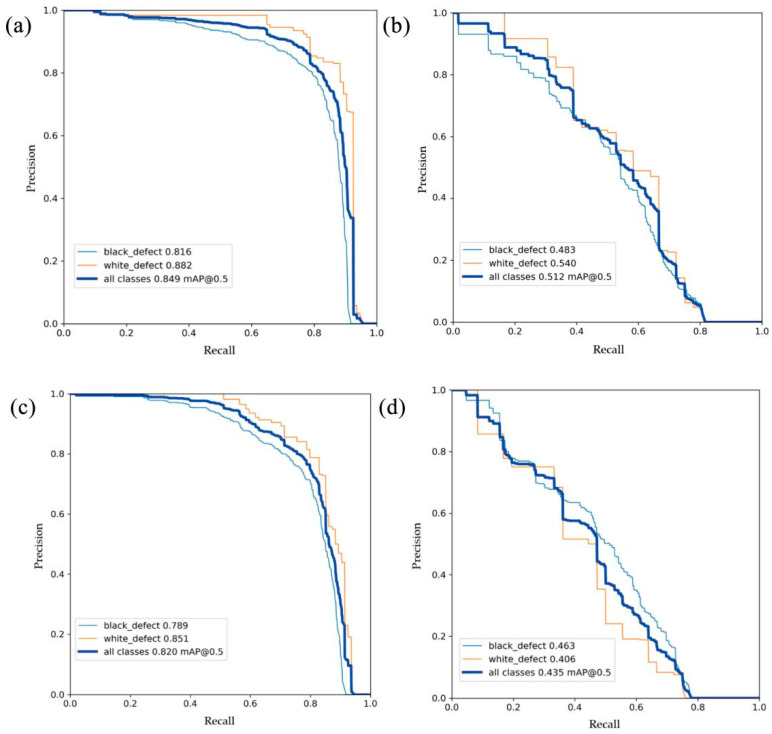
Model precision–recall curve. (**a**) PR curve for the training dataset of the preprocessed image; (**b**) PR curve for the test dataset of the preprocessed image; (**c**) PR curve for the training dataset of the typical preprocessed image; (**d**) PR curve for the test dataset of the typical preprocessed image [56].

Welding image processing technology primarily consists of three core steps: preprocessing, edge contour extraction, and feature calculation. Image preprocessing is the initial stage of molten pool image processing, with the primary objectives of eliminating image noise, enhancing molten pool edge features, and improving image contrast to lay the foundation for subsequent contour extraction and feature parameter calculation. Current mainstream image preprocessing techniques include filtering and noise reduction, image enhancement, and geometric correction. Yun et al. [56] developed an image preprocessing method that integrates defect features by analyzing pixel values. After maximizing the contrast between defects and the background using Contrast-Limited Adaptive Histogram Equalization (CLAHE), the process sequentially included denoising, thresholding, and concatenation. Comparison of algorithm performance on original images, preprocessed images (Figure 26a,b), and typical preprocessed images (Figure 26c,d) demonstrated the improvement in detection performance achieved through this preprocessing. The Mean Average Precision (MAP) values for training and test datasets were 84.9% and 51.2%, respectively, while the typical preprocessed image learning models achieved 82.0% and 43.5%, compared to 78.0% and 40.8% for the original image learning models.

**Figure 27:**
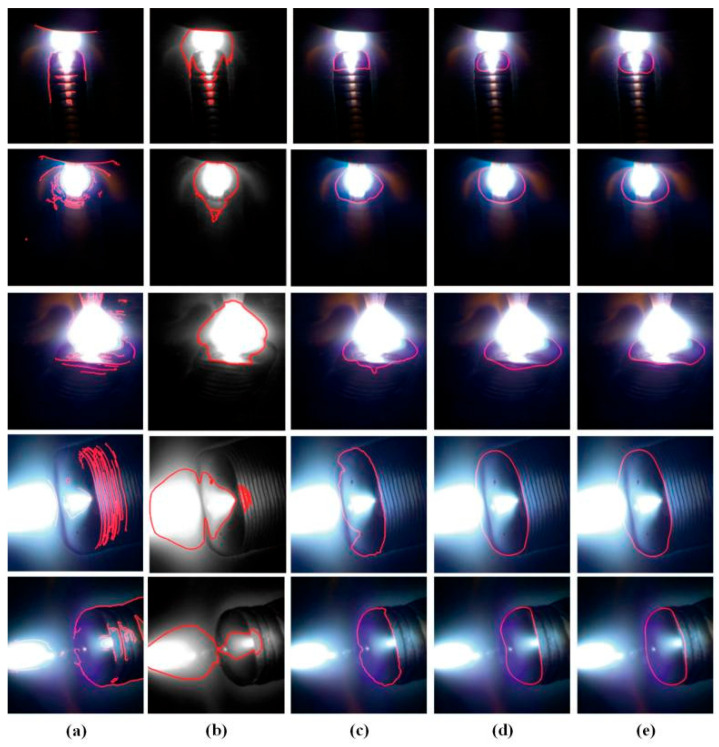
Test result of test data set. (**a**) Canny; (**b**) Chan–Vese (CV) model; (**c**) Enet; (**d**) Res-Seg; (**e**) Res-Seg + DCGAN [57].

Molten pool edge contour extraction is the core step in molten pool image processing, aiming to accurately extract edge contours from preprocessed images, thereby providing a foundation for calculating feature parameters and enabling dynamic tracking. Current melt pool contour extraction techniques primarily fall into two categories: traditional threshold-based and edge detection methods, and deep learning-based approaches. In this study, Wang et al. [57] established a dataset using melt pool images captured by visual sensing systems, designed a multi-scale feature fusion semantic segmentation network called Res-Seg, and supplemented the melt pool dataset with Deep Convolutional Generative Adversarial Network (DCGAN). Color and shape data were enhanced prior to network training. Experimental results shown in Figure 27 demonstrate that both traditional edge extraction algorithms (Figure 27a,b) and the convolutional neural network-based contour extraction method (Figure 27c–e) produce smooth and complete melt pool contours closely resembling real boundaries. Comparison between Figure 27c and Figure 27d reveals that Res-Seg yields more accurate contours than ENet, primarily due to its greater network depth, which enables the extraction of deeper semantic information during downsampling. When integrated with shallowly extracted image details, Res-Seg exhibits enhanced sensitivity to melt pool position, shape, and edge characteristics. As illustrated in Figure 27d,e, the dataset expansion strategy utilizing DCGAN further improves contour extraction accuracy.

Furthermore, Liu et al. [58] proposed a fusion pool edge detection method that combines dark channel dehazing (DCPD) with an improved single-scale Retinex image enhancement algorithm. This approach addresses the excessive edge noise in original fusion pool images and the challenges in feature extraction caused by dark regions in the fused pool after DCPD processing. Through comparative and ablation experiments, they demonstrated that the algorithm significantly enhances both image enhancement performance and feature extraction accuracy, enabling precise and complete extraction of molten pool contours.

**Figure 28:**
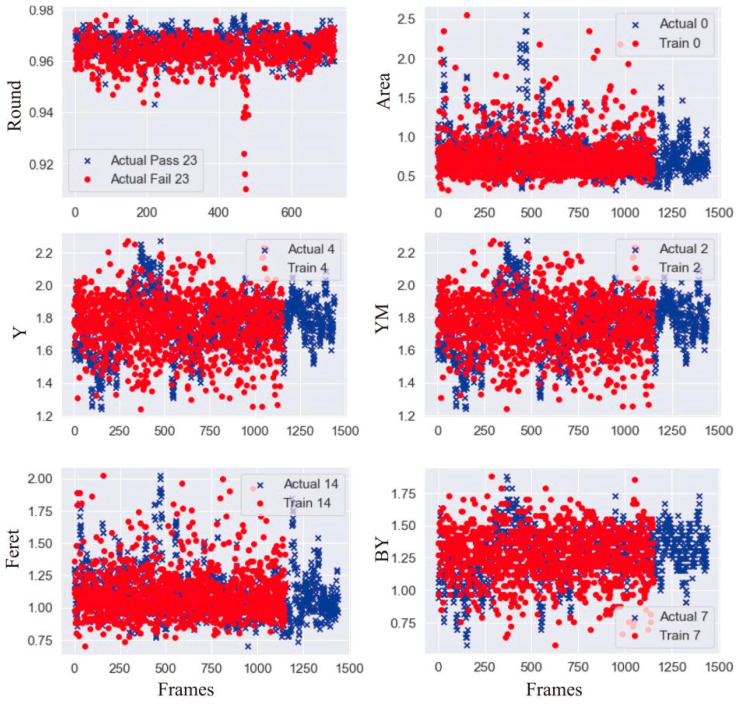
Extracted geometric features: Actual vs. predictive model [59].

The calculation of melt pool characteristic parameters in arc additive manufacturing represents the ultimate objective of melt pool image processing. By analyzing the extracted melt pool contours, key parameters reflecting melt pool status and forming quality are derived, primarily including geometric parameters (length, width, height, area, roundness, etc.) and thermal parameters (maximum temperature, average temperature, temperature gradient, etc.). To investigate porosity during welding, Aminzadeh et al. [59] employed six critical geometric features of the melt pool: roundness, area, geometric center coordinate (Y), vertical coordinate of centroid distribution (YM), Feret diameter, and vertical distance from the upper-left corner of the minimum bounding rectangle (BY), as inputs for machine learning (random forest classifier training) to characterize melt pool behavior. As shown in Figure 28, the predicted values cluster closely around the actual values, indicating that the model effectively captures the temporal dynamics of these features. Occasional dispersion likely reflects significant fluctuations in feature values or reference system effects due to overlapping category characteristics. This integration of statistical and visual evaluation demonstrates that the random forest model trained with geometric and thermal imaging features enables accurate porosity prediction and transparent interpretation.

#### 3.2.3. Multi-Modal Fusion of Visible and Infrared Data

To address issues such as low geometric accuracy and product reliability in arc additive manufacturing of large metal components, Yuan et al. [60] proposed a lightweight and efficient online control framework that integrates real-time molten pool image analysis with data-driven intelligent feedback control strategies. A single-camera vision module was developed to extract geometric features from molten pool images, enabling precise estimation of welding bead width and layer height. Experimental results demonstrate that using a single molten pool camera reduces system complexity, particularly under complex trajectories and restricted deposition angles, while avoiding the spatial and integration challenges common to multi-sensor vision systems. By integrating VQVAE, CNN, centroid position extraction, and polynomial regression techniques, the real-time molten pool images allow accurate measurement of droplet width and deposition layer height, achieving higher manufacturing efficiency and precision.

## 4. Visual Sensing-Based Defect Detection in Arc Additive Manufacturing

The forming process in arc additive manufacturing involves complex multi-physics field coupling, posing significant challenges to both form quality and structural performance. Image processing technology serves as the core foundation for ensuring manufacturing quality, with machine learning, transfer learning, and deep learning playing pivotal roles. Simultaneously, various defects during production must be accurately identified and controlled through these technologies, forming a synergistic “technology empowerment–defect mitigation” framework. However, the arc additive manufacturing process involves non-equilibrium thermal cycles and layer by layer stacking mechanisms, introducing inherent uncertainties in process stability and part quality. Consequently, anomaly detection is critical for quality monitoring. With the advancement of deep learning technologies, their deep neural network architectures enable the revelation of complex relationships among “materials–process–structure–performance” through architectural innovation, data engineering, and collaborative optimization, providing robust support for processing multi-source heterogeneous big data.

**Figure 29:**
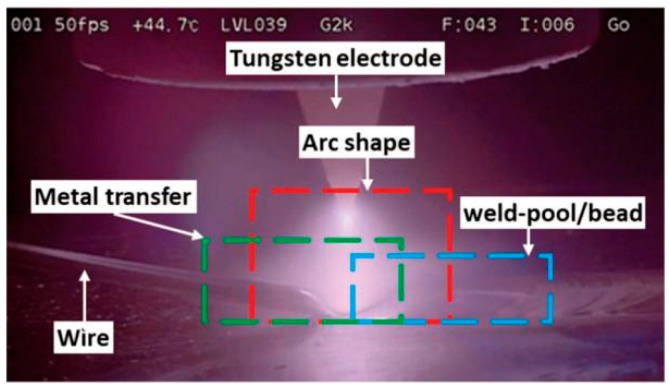
Feature information extracted by HDR camera [61].

Budiakivska et al. [61] employed three-dimensional digital image processing technology to determine optimal process parameters by measuring the strain during the stepwise removal of samples fixed on a substrate. However, most related studies applied machine learning to data-driven models for defect detection through feature and pattern learning. This approach requires substantial data acquisition, which is time-consuming and resource-intensive, posing challenges for machine learning-based abnormal defect detection. Shin et al. [62] proposed a multi-source transfer learning method to develop an anomaly detection model for spherical defect identification, thereby ensuring quality monitoring in arc additive manufacturing. The method utilizes convolutional neural network models to extract sufficient image features from multiple source materials (as shown in Figure 29), then transfers and fine-tunes the model for anomaly detection in target materials. Finally, employing a stepwise learning approach, it sequentially extracts image features from individual source materials and combines them with features from target materials to distinguish images into normal and abnormal states, achieving precise defect detection.

**Figure 30:**
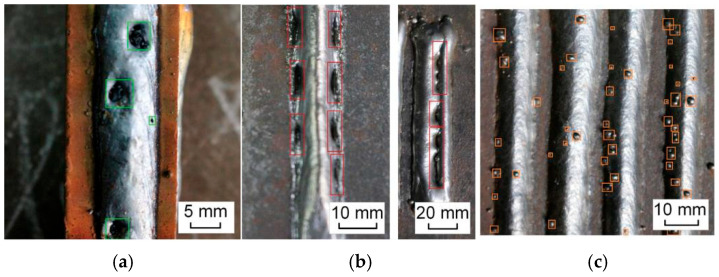
Detection of deposition defects in different materials; (**a**) surface porosity; (**b**) groove defect; (**c**) slag inclusion [63].

Furthermore, although machine learning dominates defect detection in arc additive manufacturing, the diversity of defect types and their complex formation mechanisms pose significant challenges for traditional machine learning algorithms. Li et al. [63] proposed a novel deep learning-based automatic defect detection solution using YOLOv4, which enhances three key components of the YOLO–attention model: the channel-layered attention mechanism, multi-space pyramid pooling, and exponential moving average. This approach enables rapid and accurate defect detection in arc additive manufacturing with a single application of YOLO–attention, as illustrated in Figure 30. Slag inclusions, surface pores, and grooves vary in shape and size and lack any common characteristics that would enable their distinction and localization. The YOLO–attention method effectively identifies various deposition defects and has demonstrated feasibility for practical industrial applications, making it suitable as a real-time defect detection system for arc additive manufacturing processes.

**Figure 31:**
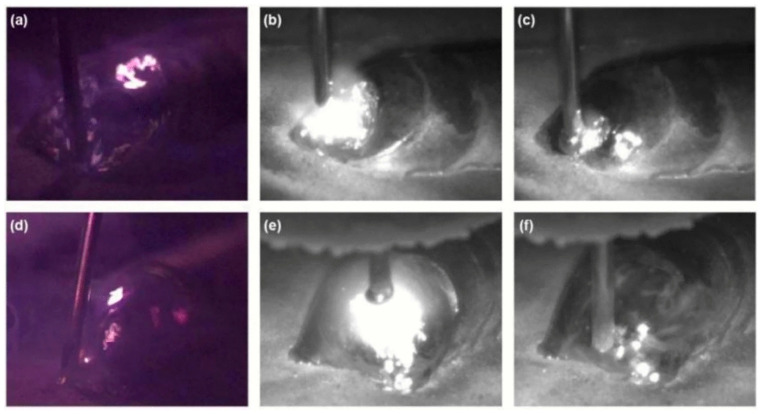
Result of molten pool imaging using passive (**a**)and active (**b**–**f**) visual camera [64].

Zhang et al. [64] proposed a multi-band visual imaging-based solution to address the challenge of melt pool imaging during arc additive manufacturing, caused by high thermal conductivity and rapid cooling rates. The method determines the radiation characteristics of the arc and melt pool through spectral analysis, employing a filter with a central wavelength of 532 nm and a bandwidth of 490 to 550 nm to suppress arc interference. With camera parameters set to a gain of 2 and exposure time of 1000 μs, the optimal melt pool edge profile is obtained, as shown in Figure 31. The optimal configuration was achieved with a gain of 2, an exposure time of 1500 μs, and a fully opened aperture (f-number = 1.4). This setup maximizes light intake while maintaining acceptably low electronic noise, resulting in a clear molten pool image with well-defined contrast between molten and solidified regions, as shown in Figure 31a. As illustrated in Figure 31b–f, the active vision system successfully captures clear images of the molten pools of both AZ91 and Mg–Gd–Y–Zr alloys during both arc combustion and arc extinction stages. This represents a significant improvement over passive methods, offering a continuous data stream for process monitoring.

**Figure 32:**
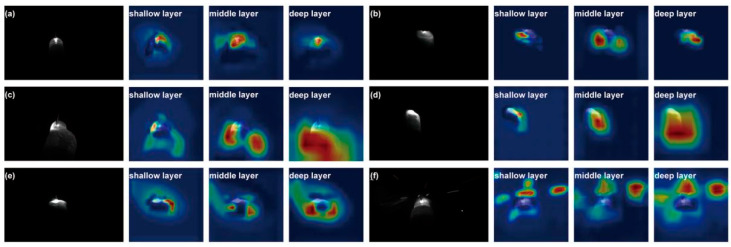
Visualization of the molten pool image features for different layers of the network. (**a**) Normal stack; (**b**) Normal overlapping; (**c**) Collapse; (**d**) OD too short; (**e**) OD too long; (**f**) spattering [65].

To address issues such as welding defects caused by inappropriate process parameters in arc additive manufacturing, Zhang et al. [65] developed an early warning system for the molten pool using deep learning methods to account for the irregularities in the manufacturing process. They constructed a molten pool image classification model employing four convolutional neural networks: VGG16, ResNet50, EfficientNetB1, and ResNet50, and performed visual analysis of the model using the Grad-CAM algorithm (Figure 32), achieving the highest classification accuracy of 98.6%. By accurately identifying the liquid zone, semi-solid zone, and solidified zone of the molten pool, the study validated the rationality of the molten pool state during arc additive manufacturing.

**Figure 33:**
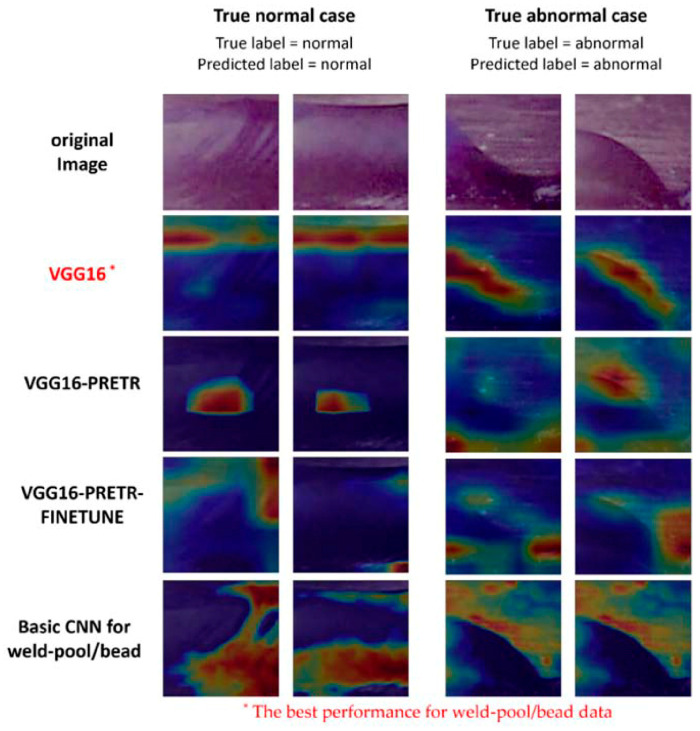
Visual verification by Grad-CAM for weld-pool/bead [66].

Due to the complexity of the arc additive manufacturing process, numerous defects during production are difficult to detect visually. To address this, Lee et al. [66] proposed using images captured by high dynamic range infrared cameras for defect detection in arc additive manufacturing. The method first preprocesses the collected images and employs an artificial intelligence model to distinguish between normal and abnormal behaviors during the manufacturing process. However, given the limited dataset of defect images, a transfer learning model (VGG16-PRETR) was employed. For model validation, the gradient-weighted class activation mapping algorithm was used to select the model with the most accurate classification performance. As shown in Figure 33, VGG16 successfully classified both spherical and edge feature information, while VGG16-PRETR demonstrated superior performance in classifying original images, effectively identifying anomalies within them.

## 5. Conclusions

Based on the comprehensive review of wire arc additive manufacturing (WAAM), the following four conclusions are drawn, covering trajectory planning, image processing, seam tracking, and their integration for quality control.

(1) Trajectory planning significantly determines the geometric accuracy and structural integrity of WAAM components, yet critical challenges remain in handling sharp corners, start/end points, and thermal accumulation. Current research demonstrates that conventional path strategies often lead to self-overlap, underfill, humping, and cumulative deformation at corners and intersections. Advanced solutions such as the spherical cap model (CCM), modified corner paths with artificial neural networks, Adaptive Process Control (APCS), and End-Lateral Extension (ELE) strategies have effectively reduced these defects. For thin-walled and thick-walled structures, region-based slicing (e.g., CL-WAAM) and composite filling algorithms (zigzag plus contour offset) mitigate stair-stepping effects and improve surface flatness. However, heat accumulation and residual stress remain tightly coupled with deposition sequence; weaving patterns and short-segment strategies yield lower residual stress and more uniform deformation compared to long or spiral paths. Furthermore, start/end defects are alleviated by return-path strategies and water-pouring sequential planning. Overall, while multi-strategy hybrid path planning has progressed, full automation that adapts to real-time thermal feedback is still lacking.

(2) Visual and infrared imaging techniques have become essential for in situ monitoring of the melt pool and weld seam, but image quality is often compromised by intense arc light, spatter, and high dynamic range. Passive vision systems using CCD or CMOS cameras, combined with adaptive thresholding, Sobel edge detection, and ROI definition, have enabled real-time extraction of bead width, centerline deviation, and wire deflection. For example, binocular prism systems achieve layer width root-mean-square errors below 0.2 mm. Infrared thermography captures temperature fields and heat accumulation, revealing that interlayer temperature rises rapidly in the first few layers before reaching equilibrium. To overcome arc interference, multi-modal fusion networks (e.g., MMFNet) combining visible and infrared data achieve over 98% prediction accuracy. Deep learning models such as YOLOv26, Res-Seg, and DCGAN-augmented segmentation have improved melt pool contour extraction, while semi-automated annotation tools (SAM + XMem) accelerate video analysis. Nevertheless, robust feature extraction under highly reflective materials (aluminum, copper) and complex curved surfaces still requires further development of multi-spectral or polarimetric imaging.

(3) Real-time seam tracking and defect detection have been greatly enhanced by machine learning and deep learning, yet data scarcity and model generalization remain major hurdles. Supervised learning approaches, including random forests for porosity prediction (using geometric features like roundness, area, Feret diameter) and CNNs (VGG16, ResNet50, EfficientNet) for molten pool classification, have achieved classification accuracies above 98%. For defect detection, YOLO–attention variants (channel attention, spatial pyramid pooling, EMA) rapidly identify surface pores, groove marks, and slag inclusions. Transfer learning (e.g., VGG16-pretrained) and multi-source transfer learning have been employed to detect balling defects when target-material datasets are small. However, the review highlights that most current models are material- and geometry-specific, and the lack of large, labeled WAAM process datasets limits the deployment of generalizable anomaly detectors. Moreover, real-time closed-loop control based on vision feedback is still primarily demonstrated for layer width regulation, while full integration with adaptive trajectory replanning remains an open challenge.

(4) Despite significant advances in trajectory planning and image-based monitoring, the integration of these technologies into a unified closed-loop control system is still immature, and future efforts must focus on real-time data fusion and predictive modeling. Existing studies treat path planning (offline) and process monitoring (online) largely separately. While Adaptive Process Control adjusts welding speed and wire feed rate based on preset segments, and feedback control regulates layer width using passive vision, there is no seamless framework that continuously updates the deposition path from melt pool images or thermal fields. The review identifies key gaps: (i) trajectory algorithms do not compensate for thermal distortion in real time; (ii) most image processing is post-processing rather than real-time control; (iii) residual stress and deformation are simulated offline but rarely used for on-the-fly correction. Promising directions include AI-driven trajectory optimization (e.g., GNNs (Graph Neural Networks) or RL (Reinforcement Learning)) and digital twins that synchronize physical and virtual models. In summary, the path toward intelligent WAAM requires merging adaptive slicing, online defect detection, and thermal-mechanical simulation into a single, sensor-driven autonomous system.

(5) Future research should integrate artificial intelligence, digital twins, advanced imaging, and reinforcement learning to overcome key limitations in wire arc additive manufacturing. Specifically, deep learning frameworks combining graph neural networks or transformers with self-supervised learning from high-speed images can enable adaptive trajectory planning and real-time melt pool anomaly prediction. A high-fidelity digital twin, updated at over 50 Hz and driven by multi-sensor data assimilation, would allow predictive control via synchronized thermal-fluid simulation. To improve feature extraction under intense arc light, multi-spectral and polarimetric imaging should be explored, leveraging hardware acceleration for real-time processing. Finally, model-free reinforcement learning algorithms, such as soft actor–critic or proximal policy optimization, can continuously correct trajectory drift caused by thermal distortion by mapping visual features directly to robot adjustments. Key challenges remain in acquiring labeled datasets, ensuring generalizability across materials and geometries, reducing computational costs through surrogate models or edge computing, and designing efficient training and reward mechanisms. Addressing these fronts will enable closed-loop, autonomous WAAM systems with enhanced accuracy, reliability, and adaptability for large-scale metallic components.

## Figures and Tables

**Table 1:** Comparison of representative trajectory planning methods for WAAM.

Method Category	Representative Work	Applicable Geometry	Key Advantages	Main Limitations
Corner optimization	Ding et al. [23]	Thin-walled structures with sharp corners	Reduces self-overlap at corners	Primarily designed for aluminum; limited validation on other materials
Modified corner path with ANN	Ding et al. [24]	Gap-free thick-walled structures	Avoids both overfill and underfill at corners	Requires extensive training data; geometry specific
Adaptive process control	Li et al. [25]	Complex-shaped components with high curvature	Matches welding speed and wire feed rate dynamically	Increased computational complexity
End-lateral extension	Li et al. [27]	Components with path intersections	Eliminates intersection defects; improves interlayer consistency	Best suited for specific intersection geometries
Curved layer WAAM	Hu et al. [33]	Curved and freeform surfaces	Mitigates step effect; better surface conformity	More complex path generation; longer computation time
Composite filling	Zhang et al. [34]	Components requiring high contour accuracy	Optimizes residual stress distribution; improves contour accuracy	Parameter tuning required for different geometries
Thermal behavior aware planning	Zhao et al. [35]	Large shell-shaped components	Reduces interlayer temperature gradients; improves dimensional accuracy	Computationally intensive finite element simulations
Enhanced overlapping model	Li et al. [36]	Multi-layer multi-bead metallic parts	Improves surface flatness; reduces internal defects	Relies on accurate ANN predictions

## Data Availability

No new data were created or analyzed in this study.
